# Motivations To Join And Stay A Member Of A Village For Older Adults

**DOI:** 10.1093/geroni/igab046.2369

**Published:** 2021-12-17

**Authors:** Joonyoung Cho, Ruth Dunkle, Garrett Pace

**Affiliations:** University of Michigan, Ann Arbor, Michigan, United States

## Abstract

People join a customer-driven organization with motivations that may not be static over time, an important issue for long-term organizational viability. In this study, we examined motivations among members of ShareCare, the first Village for older adults in the U.S. Using qualitative data from a random sample of 91 members, we compared motivations for becoming a member and for continuing membership. Motivations to join and continue membership are not necessarily the same. Motivations were categorized as: instrumental, social, and altruistic motivation. We categorized length of membership as short-term: 8-years or less (51.63%) and long-term: 9-years and more (49.37%). While 36% of members joined only for instrumental motivation, 59% continued membership only for instrumental motivation. While about 52% joined with multiple motivations, only 35% of members mentioned multiple motivations when continuing their membership. Finally, 18% of short-term members mentioned altruistic motivation when continuing their membership, while 28% of long-term members mentioned altruistic motivation when continuing their membership. While people’s motivation might change over time, altruistic motivation may be the greatest motivating factor for long-term memberships. Long-term members may identify themselves as supporters rather than users of the organization and cultivate stronger connections with other members over time. Our findings inform how to recruit and retain members in Villages, and customer-driven organizations for older adults more broadly.

